# Developmental disturbances in early life stage mortality (M74) of Baltic salmon fry as studied by changes in gene expression

**DOI:** 10.1186/1471-2164-7-56

**Published:** 2006-03-17

**Authors:** Kristiina AM Vuori, Heikki Koskinen, Aleksei Krasnov, Paula Koivumäki, Sergey Afanasyev, Pekka J Vuorinen, Mikko Nikinmaa

**Affiliations:** 1 Centre of Exellence in Evolutionary Genetics and Physiology, Department of Biology, University of Turku, FI-20014 Turku, Finland; 2 Institute of Applied Biotechnology, University of Kuopio, P.O.B. 1627, 70211 Kuopio, Finland; 3 Sechenov Institute of Evolutionary Physiology and Biochemistry, M.Toreza av. 44, Petersburg, 194223, Russia; 4 Finnish Game and Fisheries Research Institute, P.O. Box 2, FI-00791 Helsinki, Finland

## Abstract

**Background:**

We have studied alterations of gene expression associated with naturally-occurring early life stage mortality (M74) in Baltic salmon using a cDNA microarray and real time PCR. M74-affected fry have several typical neurological, cardiovascular and pathological symptoms. They are also characterized by low thiamine content and show signs of oxidative stress.

**Results:**

Affected fry can be divided into three major groups with early, intermediate or late onset of mortality. If mortality starts during the first third of the yolk-sac stage, virtually all the responses are compatible with stress, which rapidly leads to the common terminal responses. If death occurs during the second third of the yolk sac stage, the terminal stage is preceded by a decrease in globin gene expression, which leads to internal hypoxia when the animals grow and shift from skin- to gill-breathing. Fry will eventually proceed to the terminal responses. The group developing M74 most slowly appears to compensate for reduced oxygen delivery by downregulation of metabolism, and hence some fry can escape death.

**Conclusion:**

Our study is the first demonstration of diverse transcriptional responses to a naturally-occurring developmental disturbance. Since many of the genes differentially expressed in M74-fry are evolutionarily conserved, the M74 of Baltic salmon can serve as a model for developmental disturbances and environmental stress responses in vertebrates in general.

## Background

The study of developmental disturbances using mammals is complicated because of the internal development of the embryo. The zebrafish has become an important model organism in developmental biology because of its external fertilization and development, and the ease by which embryos can be manipulated by controlling the water composition. However, studies on environmentally relevant developmental disturbances are facilitated if some natural populations of animals are characterized by such disturbances. Early life stage mortality, the death of fish during the yolk-sac stage, i.e. during development, is a common response to exposure to stressful environmental conditions. For example, Atlantic salmon (*Salmo salar*) in the Baltic Sea suffers from abnormally high, maternally-transmitted yolk-sac fry mortality (designated M74; [1]). In the 1990s, 50–90% of newly-hatched salmon from wild parents in Sweden and Finland died during the yolk-sac phase [2,3]. There are also indications that M74 has affected natural spawning populations in Swedish rivers during years of high incidence [4]. Although the syndrome is characterized by the low thiamine content of the affected fry and can be treated by addition of thiamine [5,6], the proximal cause of the syndrome has remained a mystery. As possible causes, environmental toxins, especially dioxin equivalents, algal blooms and changes in the food of Baltic salmon have been advocated.

M74-affected fry have several neurological, cardiovascular, morphological and other symptoms such as disturbed swimming pattern, impaired coordination and lack of phototaxis, decreased heart rate, decreased yolk absorption, a small and pale spleen, blood congestion, reduced number of circulating erythrocytes, abnormal haemorrhages/blood coagulation and a high frequency of necrotic cells in the brain [1,7,8]. Dying yolk-sac fry are lethargic, and have convulsions and bradycardia [1,7].

Many pathological findings in M74 are compatible with disturbances in the cellular redox state, particularly long-term oxidative stress. Eggs and fry that subsequently develop M74 are characterized by low thiamine content [5]. The symptoms of M74 can be treated with thiamine and partly induced by thiamine antagonists [6]. M74 is associated with decreased levels of antioxidants such as astaxanthin, α-tocopherol and ubiquinone [9,10]. The cellular glutathione ratio (GSH/GSSG) is altered in favour of the oxidized form [11], and the activities of liver redox enzymes – glutathione peroxidase, glutathione reductase and glutathione-S-transferase – are increased [11,12]. Moreover, M74 fry have more oxidized fatty acids than healthy fry [13].

The multiple symptoms observed in the developmental disturbances of salmon indicate that many genes are affected. Expression of a large number of genes can be analyzed using cDNA microarrays. In this study we have used a salmonid cDNA microarray enriched with stress genes [14] to study gene expression changes associated with the M74 developmental disturbance and associated mortality. Our study is the first demonstration of altered transcriptional responses associated with naturally-occurring early life stage mortality. Many genes show changed expression patterns before the fry manifest any symptoms of M74. Since many evolutionarily conserved stress-inducible genes (i.e. [15-19]) appear to be affected, this naturally-occurring developmental disturbance may indicate some of the general pathways that can be affected during development of vertebrates subjected to environmental stresses.

## Results and discussion

**Figure 1:**
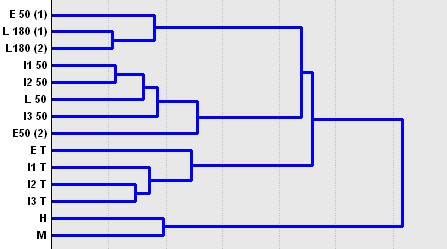
**Classification of M74 groups and stages by gene expression**. For the explanation of sample groups, see Table I. Euclidian distances between log_2_(ER) values were clustered using Ward's method. H denotes healthy fry 50 vs 180 ATU and M denotes M74 (I1 and I2) 50 vs 180 ATU hybridizations. The other hybridizations are M74 vs healthy fry of the same age.

**Figure 2:**
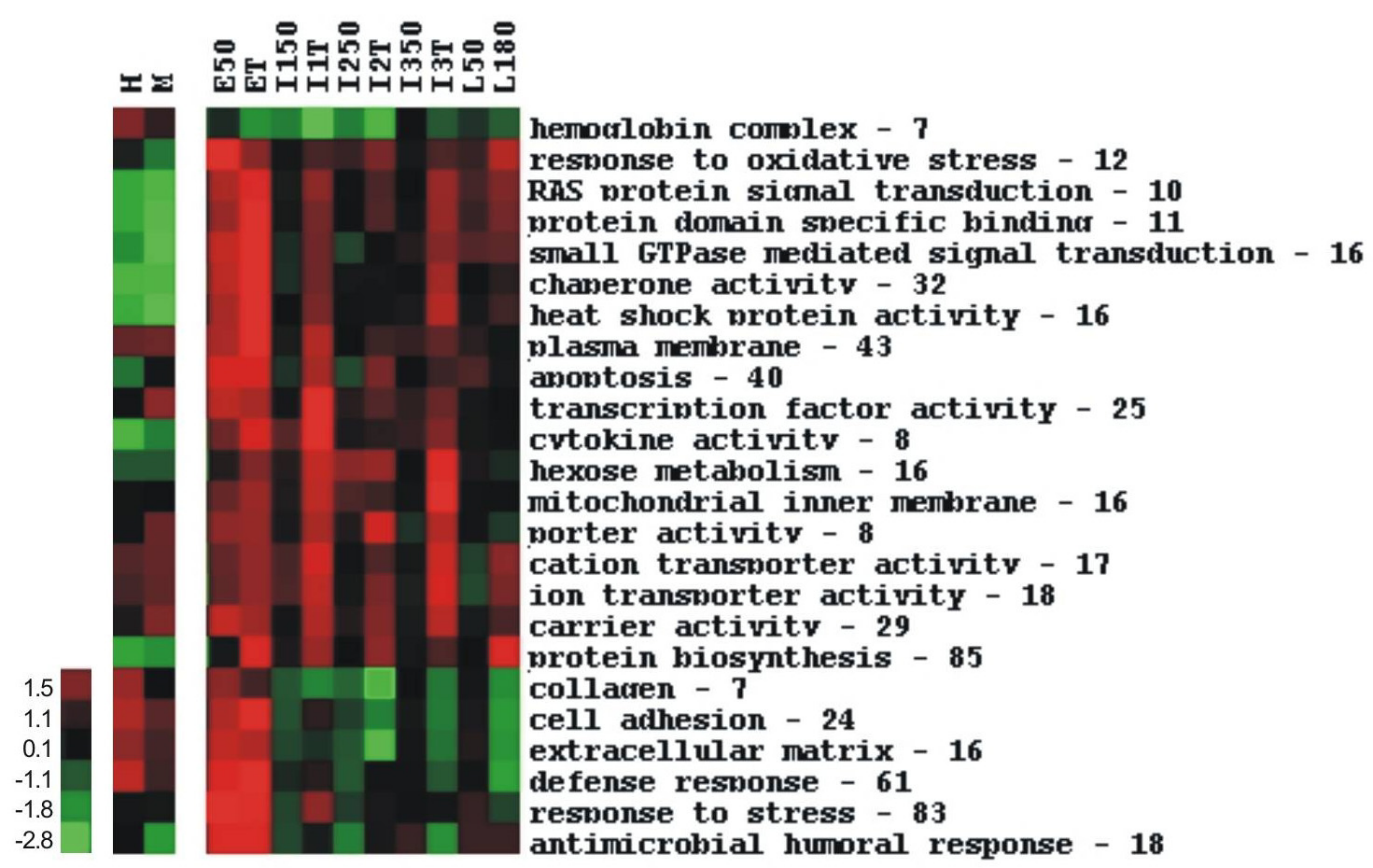
**Classification of different M74 groups and stages by Gene Ontology functional classes**. For explanation of sample groups, see Table I. H and M are shown separately owing to different hybridization designs: H denotes healthy fry 50 vs 180 ATU and M denotes M74 (I1 and I2) 50 vs 180 ATU hybridizations. The other hybridizations are M74 vs healthy fry of the same age. Differentially expressed genes were grouped by the Gene Ontology categories and mean log_2 _(ER) were analyzed by ANOVA and the Newman-Keuls test. The panel presents functional classes including at least 5 genes that differed significantly between groups (p < 0.05). The mean log_2 _expression values are colour-coded; red denotes upregulation and green downregulation. The numbers of genes per GO class are indicated.

The embryos developing M74 were divided in three major groups (E – early, I -intermediate and L – late) on the basis of the onset of the developmental disturbances; the I group included 3 subgroups (Table 1). Samples were taken at different times to encompass the preclinical, clinical and terminal stages (Table 1). At the preclinical stage, no visible symptoms of M74 are seen. At the clinical stage, M74-fry have various typical, i.e., neurological and cardiovascular, symptoms. The terminally ill fry are lethargic and have convulsions in addition to the clinical symptoms [1,7]. Cluster analysis of gene expression data placed all terminal and preclinical stages in separate clusters (Fig. 1), whereas the data from the clinical stages were divided. The biological variability between clinical stages in the E- group may be higher than in the L-group, which can be accounted for by the rapid progression of M74 and the severe condition of the fry. The between-groups similarities and differences in GO functional classes are shown in Fig. 2. Several genes in such functional classes as 'response to oxidative stress' and 'apoptosis' are upregulated at all M74 terminal stages and in the E-group. In contrast, genes in the functional classes 'collagen', 'cell adhesion' and 'extracellular matrix' are upregulated in the E-group but downregulated in other M74 groups.

In most analyses, M74-affected vs healthy fry of same age were compared. However, we also performed two additional microarray hybridizations in which 50 vs 180 ATU healthy fry, and 50 vs 180 ATU M74 fry, were compared. Cluster analysis (Fig. 1) showed that age-related changes in both healthy and M74 fry (50 vs 180 ATU comparisons) were clearly different from M74 vs healthy comparisons of same age. Although the directions of changes in the 50 vs 180 ATU hybridizations were largely similar in both healthy and M74 specimens, there were differences in the magnitude of up/downregulation of many genes.

### Common genes affected throughout the developmental disturbance include those coding for globin chains and histone H1.2

**Figure 3:**
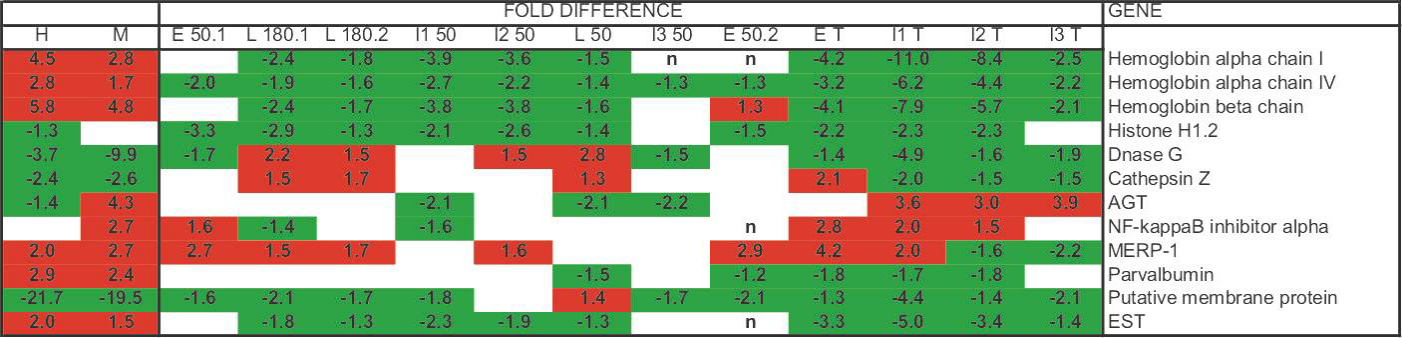
**Common alterations of gene expression in M74 groups**. The sample groups are in the same order as in the clustergram in Fig. 1. For explanation of sample groups, see Table I. H and M are shown separately owing to different hybridization designs: H denotes healthy fry 50 vs 180 ATU and M denotes M74 (I1 and I2) 50 vs 180 ATU hybridizations. The other hybridizations are M74 vs healthy fry of the same age. The panel presents genes that were differentially expressed in at least 5 of 10 samples. Red indicates upregulation and green downregulation. The values are expressed as fold difference. White means that the expression ratio did not significantly deviate from unity and n means the absence of data.

Genes that were differentially expressed throughout M74 are presented in Fig. 3.

**Figure 4:**
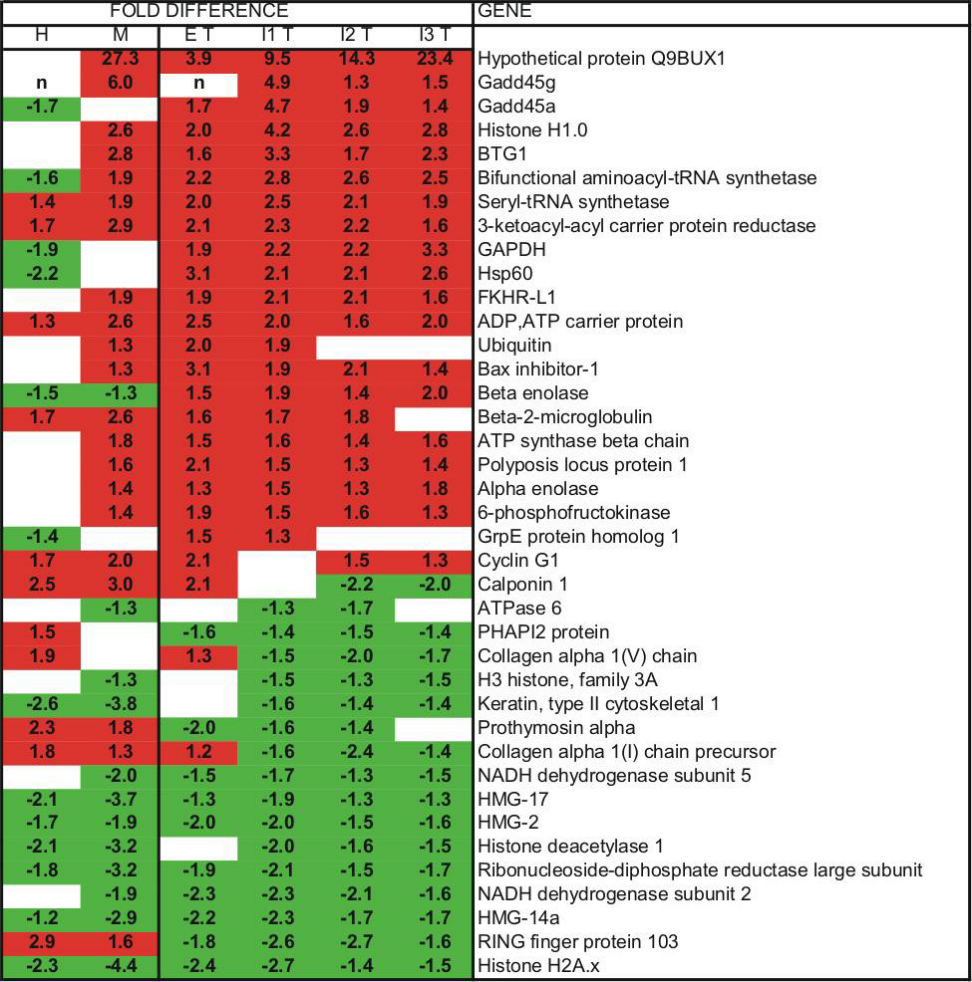
**Gene expression at the terminal stages of M74**. The panel presents genes that were differentially expressed in at least 3 of the 5 groups. For explanation of the sample groups, see Table I. H denotes healthy fry 50 vs 180 ATU and M denotes M74 (I1 and I2) 50 vs 180 ATU hybridizations. The other hybridizations are M74 vs healthy fry of the same age. Red indicates upregulation and green downregulation. The values are expressed as fold difference. White means that the expression ratio did not significantly deviate from unity and n means the absence of data.

Two genes with unknown function, a putative membrane protein (Q98SE3) and an unidentified EST, are downregulated at all stages of M74. With regard to genes with known function, M74 fry already suffer from downregulation of genes encoding the globin chains of adult type haemoglobin during the preclinical stages of the syndrome, before any visible symptoms are observed. Notably, expression of the adult globin genes increases between 50 and 180 ATU in normal development. This increase is blunted between 50 and 180 ATU during development of M74-affected fry (Figures 3 and 4). Since haemoglobin synthesis requires initial formation of globins and recruitment of haem moieties to preformed globin chains, reduced adult type globin gene expression can account for the typical symptoms of M74-affected fry, i.e., pale blood, spleen, liver and gills. The period of haemoglobin (and red blood cell) switching from embryonal to adult types occurs after hatching [20]. Notably, M74-affected fry dying at the intermediate period of yolk-sac fry development (180–200 ATU) show reduced DNA-binding of HIF1-α [21]. In mammals, HIF1-α regulates erythrocyte formation [22]. Reduced haemoglobin synthesis leads to a reduced blood haemoglobin concentration causing reduced oxygen delivery and consequent hypoxia. Hypoxia is teratogenic to zebrafish embryo, influencing, e.g., apoptosis [23]. Thus, disturbances such as decreased cell proliferation and increased cell death, which are observed in the terminal stages of M74, may to some extent be the ultimate result of reduced oxygen transport due to reduced haemoglobin production.

Histone H1.2 is also downregulated at different stages and in different groups of M74 fry. Histone H1.2 is a linker found in most somatic cell nuclei. Its expression is coupled to the cell cycle – it is synthesized only during S-phase [24]. Downregulation of H1.2 indicates that the rate of cell proliferation has already decreased in the preclinical stages of the syndrome.

Some genes (DNase γ, cathepsin z, NF-kappaB, MERP-1) changed expression in all M74-groups but in different directions. One of the general responses observed in various forms of stress, such as hypoxia, is an increased rate of apoptosis (programmed cell death) or necrosis. One of the best-known changes associated with apoptosis is endonuclease activity. The cleavage of chromatin into oligonucleosomal fragments, which form the characteristic apoptotic DNA ladder, has been documented in numerous models of cell death. The DNases involved in apoptotic DNA fragmentation are considered to differ among cell types, differentiation states, and/or apoptotic stimuli. DNase γ is a member of the DNase I family. It has high activity in such haematopoietic and lymphoid organs as bone marrow, spleen, lymph nodes, thymus and liver; this is also the case in Xenopus [25]. DNase γ can produce apoptotic DNA fragmentation [26,27]. Although the DNases involved are have not been identified, DNA fragmentation may also be associated with necrosis [28]. Interestingly, DNase γ is already upregulated in the L group at the preclinical stage (50 ATU), which is far from the appearance of first symptoms, but is downregulated in the terminal stages of the disease in other M74 groups. The protease cathepsin z, which may be involved in necrosis [29], is expressed in similar manner to DNase γ. Although both necrosis- and apoptosis-like cellular changes have been reported in M74 fry [8,30], the role of the variable expression of DNase γ and cathepsin z in these cellular changes remains only speculative.

The gene for alanine-glyoxylate aminotransferase (AGT) is downregulated early in M74 groups, before any clinical symptoms of the disturbance are observed, but it is upregulated at the terminal stages of the syndrome. The fact that salmon fry depend on the yolk as their energy source offers one explanation for this observation. Disturbances in amino acid catabolism interfere with the utilization of yolk, and decreased utilisation of yolk and depletion of glycogen stores are among the symptoms observed in M74-affected fry [1,8]. AGT catalyzes the breakdown of alanine and serine to pyruvate. Thus, its downregulation may reflect a decreased utilization of yolk protein in the early stages of development, and its later upregulation an increased need for gluconeogenesis from amino acids.

Nuclear factor-κB (NFκB) is a redox-sensitive transcription factor that is activated in response to a broad range of stimuli including inflammatory cytokines, UV radiation and H_2_O_2 _[31,32]. The cytoplasmic NFκB in unstimulated cells is a transcriptionally active dimer bound to an inhibitor protein, IκB. The predominant subunits of NFκB are p50 and p65. Upon stimulation, IκB is rapidly phosphorylated and degraded. The released dimer can then translocate to the nucleus and activate target genes [31,32]. In E 50, IκBα is upregulated. This indicates that NFκB activity may be increased, because an increase in NFκB-activity causes increased IκB transcription via a negative feedback loop. In contrast, IκBα is downregulated in the L-group at 180 ATU (verified by Q-RTPCR).

Ependymin is present in the extracellular fluid (ECF) and the cerebrospinal fluid (CSF) of teleost brain. Its concentration in ECF and CSF is maintained by synthesis and secretion by specific cells. Ependymin may be involved in establishing new synaptic connections and strengthening pre-existing ones during, e.g., learning and neuronal growth [33,34]. Variations in the expression of mammalian ependymin related gene 1 (MERP-1) in M74 groups may be relevant to the development of the neurological symptoms typical of M74, which are observed especially in the terminal stages of the syndrome.

### Terminal stages of the syndrome are associated with a gene expression profile compatible with inhibition of the cell cycle and cell proliferation and consequent cell death

A panel of genes associated with stress responses, cell cycle and growth arrest, glycolytic energy production and cell death showed common expression profiles in the terminal stages of the M74 syndrome (Fig. 4).

There is a clear overall downregulation of chromatin components and an upregulation of growth arrest signals in M74 fry. In addition to H1.2, which is downregulated throughout the developmental disturbance (Fig. 3), genes coding for several of the chromatin and protein components involved in chromatin remodelling and DNA metabolism (histones H3A and H2A.x, high mobility group proteins (HMGs) -2, -14a and -17, prothymosin α, ribonucleotide reductase M1 chain, histone deacetylase 1) were similarly repressed. Upregulated growth arrest-related genes included BTG1, polyposis locus protein 1 and histone H1°.

Histones H2A.x and H1° are replacement histone subtypes. Their expression is cell cycle independent and they are synthesized in nonproliferating cells. Their gradual accumulation parallels a decrease in the main type histones of the corresponding class. In mammals, H2A.x is found at high levels in testis, thymus and spleen, and in lower amounts in ovary and intestine [24]. The downregulation of H2A.x in the terminal stages of M74 may reflect a reduced number of differentiating cells and consequent slowing down of development. In contrast, the histone H1° gene is upregulated. Notably, histone H1° was originally found only in tissues with minimal cell division, such as liver, kidney and brain. Subsequently, it was shown that the synthesis of histone H1° increases during growth inhibition [24]. Thus, upregulation of the histone H1° gene in the terminal stages of M74 is in line with the retardation of growth.

Genes for three different high mobility group proteins (HMGs), HMG-2, HMG-14a and HMG-17, are downregulated in the terminal stages of the syndrome. HMG proteins function as structural elements of chromatin, generating a conformation that facilitates and enhances various DNA-dependent activities. The amount of HMG-1/-2 in a cell is about 10-fold lower than that of a histone and the amount of HMG-14/-17 is 10-fold-lower than that of HMG-1/-2. The HMG families HMG-1/-2 and HMG-14/-17 have a unique functional motif. They induce specific conformational changes in their binding sites thus facilitating, e.g., DNA binding of transcription factors [35]. Moreover, the depletion of HMG-2 by antisense technology slows down the rate of cell proliferation [36], compatible with the suggestion that the downregulation of the gene in M74 syndrome is associated with reduced cell renewal.

The downregulation of prothymosin α (ProTα), ribonucleotide reductase M1 chain and histone deacetylase 1 (HDAC1) in terminal stages of M74 is most likely associated with reduced transcriptional activity and DNA replication. Prothymosin α is a widely-expressed highly acidic protein that induces the unfolding of chromatin fibres. This process is a prerequisite for chromatin decondensation, which is needed before transcription or DNA replication can occur [37]. Ribonucleotide reductases provide the precursors necessary for DNA synthesis and replication in all living cells. The activity of ribonucleotide reductase is closely correlated with the cell growth rate and appears to vary with the cell cycle [38]. Acetylation of chromatin is associated with active gene expression [39]. Downregulation of HDAC1 is specific to the I-groups and may indicate that repression of transcription is specifically inhibited.

That cell proliferation is reduced in terminal stages of M74 is also indicated by increased expression of the genes for the anti-proliferative polyposis locus protein 1 and BTG1. BTG1 is normally expressed early during the G0/G1 transition of the cell cycle. Its expression decreases quickly as the cells progress through the cycle. The BTG1 gene is induced when, e.g., genotoxic stress leads to cell cycle arrest [40]. In fish, BTG1 expression is upregulated in hypoxic *Gillichtys mirabilis *[41]. The yeast homologue of polyposis locus protein 1, Yop1p, regulates cell growth negatively; its overexpression may result in cell death, an accumulation of internal cell membrane, and a block of membrane traffic [42].

Imminent cell death is indicated by the upregulation of several cell stress and death associated genes in terminal stages of M74. These include the evolutionarily conserved death suppressor Bax inhibitor-1 (BI-1), the transcription factor forkhead box-like1 (FKHR-L1), the DNA-damage and repair associated genes Gadd45alpha (Gadd45a), Gadd45gamma (Gadd45g) and CyclinG1, β-2-microglobulin and the protein degradation related gene polyubiquitin UBC.

Overexpression of forkhead box protein O3A (FKHR-L1) causes growth suppression and cell cycle arrest in a variety of cell lines [43]. When the stress level is high, FKHR-L1 may also promote the cell death program [43,44]. In milder stresses, FKHR-L1 induces a delay in the G2-M transition of the cell cycle during which it helps to repair damaged DNA by a Gadd45-dependent mechanism [44]. Notably, both FKHR-L1 and Gadd45 are strongly upregulated in the terminal stages of M74, suggesting an attempt to facilitate DNA repair. Cyclin G1 is one of the target genes of the transcription factor p53, and is induced in a p53-dependent manner in response to DNA damage. It plays roles in G2/M arrest, damage recovery and growth promotion after cellular stress [45].

BI1 is predominantly colocalized in intracellular membranes with proteins of the Bcl-2 family. BI1 can interact with antiapoptotic Bcl-2 and Bcl-x and suppress apoptosis caused by Bax. It is induced in many kinds of environmental stresses such as oxidative stress, ER stress, heat shock, chemicals and growth factor deprivation from yeast and plants to man [16,46].

Cell death can be initiated by both lack of oxygen and oxidative stress [47,29]. As long as cells can maintain their reducing capacity against reactive oxygen species, apoptotic cell death occurs, whereas necrosis is triggered when the reducing homeostasis is disturbed and antioxidant defence fails [29,28]. There are several indications that the redox state of the M74-affected fry is altered. Lundström et al. [12] have reported that redox enzyme activities increase in M74 fry in clinical and terminal stages of the syndrome. At the transcriptional level, there is marked upregulation of the mRNAs for several redox enzymes and thioredoxin-like proteins only in the clinical stage in the group that manifests the syndrome earliest (data not shown). Transcripts of redox enzymes are also upregulated, although to a lesser extent, in the clinical stage of the L-group and terminal stages of the I2- and I3-groups (data not shown). Thus, transcriptional upregulation of redox enzymes possibly occurs after the existing enzymes are maximally activated in the clinical stages of the disturbance.

Mitochondrial abnormalities such as hydropic degeneration and increases in matrix density are associated with the clinical and terminal stages of M74 [30]. The increased expression of mitochondrial chaperones, 60 kDa heat shock protein (Hsp60) and GrpE protein homolog 1 (Mt-GrpE), may be related to these changes in the terminal stages. Increases in mitochondrial inner membrane proteins in M74 terminal fry were also detected by GO functional classification (Fig. 2). The expression of mitochondrial ADP/ATP translocase 2 and ATP synthase beta chain genes is also increased, and this may reflect abnormal mitochondrial function upon injury. ADP/ATP translocases also form non-specific pores in apoptotic mitochondrial membranes. Permeabilization of mitochondrial membrane is an important player in cell death [48]. Downregulation of ATPase subunits, as in hypoxic zebrafish embryos [49], is specific to the I-group, which also shows the most marked reduction in haemoglobin synthesis.

Increase of anaerobic metabolism in M74 compared to healthy fry can be seen from upregulation of the genes for several glycolytic enzymes (6-phosphofructokinase, glyceraldehyde 3-phosphate dehydrogenase, α- and β- enolases) and enrichment of the corresponding GO functional class 'hexose metabolism' (Fig. 2) at the terminal stages. A similar upregulation of glycolytic enzyme gene expression is commonly observed in hypoxia (e.g. [50]), and was also detected in a microarray study of zebrafish embryos exposed to hypoxia [49]. If glycolytic rates are increased, the carbohydrate substrates are depleted rapidly, resulting in an increased use of amino acids in glycolysis. Notably, a gene of amino acid metabolism, AGT, which is downregulated in the early stages of the syndrome, is upregulated at the terminal stage (Fig. 3).

In addition, the muscle protein gene calponin, the intermediate filament keratin and the extracellular matrix collagen are commonly downregulated in the terminal stages of M74 (Figs. 4). Notably, muscle-specific genes, keratin and collagen are also downregulated in zebrafish embryos exposed to hypoxia [49].

Several genes associated with the terminal stages of M74 have unknown functions. These include an EST similar to the zinc finger protein KF-1, a hypothetical protein Q9BUX1, and an EST similar to the fatty acid biosynthetic enzyme 3-ketoacyl-acyl carrier protein reductase. Interestingly, the hypothetical protein Q9BUX1 is one of the genes showing the most marked responses to the developmental disturbance, being upregulated up to 30 fold in the terminal stage of the syndrome. Q9BUX1 belongs to the same protein family as ChaC, which is thought to be associated with the putative ChaA Ca^2+^/H^+ ^cation transport protein of *Escherichia coli *[51].

### The group with early onset of M74 (E-group) suffers from severe stress, and the unique gene expression changes observed will rapidly lead to those generally observed in the terminal stages

**Figure 5:**
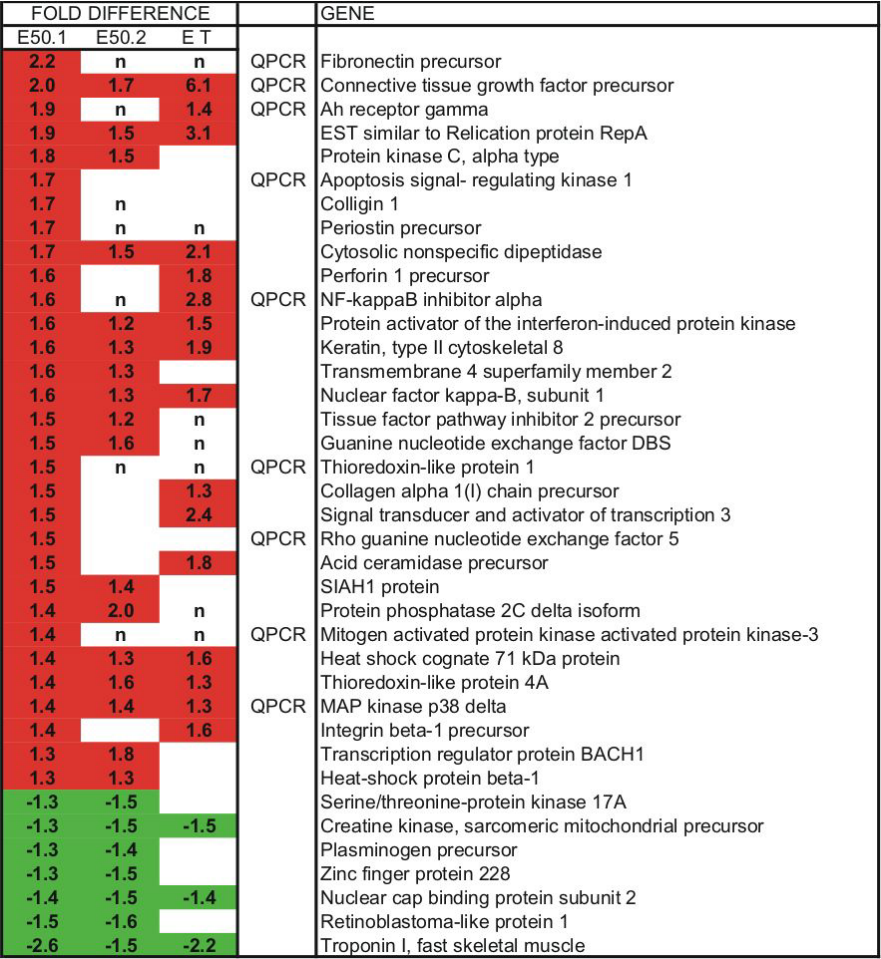
**Differentially expressed genes in Early M74-group**. E50.1 and E50.2 are biological replicates. All hybridizations are M74 vs healthy fry of the same age. Red indicates upregulation and green downregulation. The values are expressed as fold difference. White means that the expression ratio did not significantly deviate from unity and n means the absence of data. The genes analyzed by Q-RTPCR are marked in a separate column.

**Figure 6:**
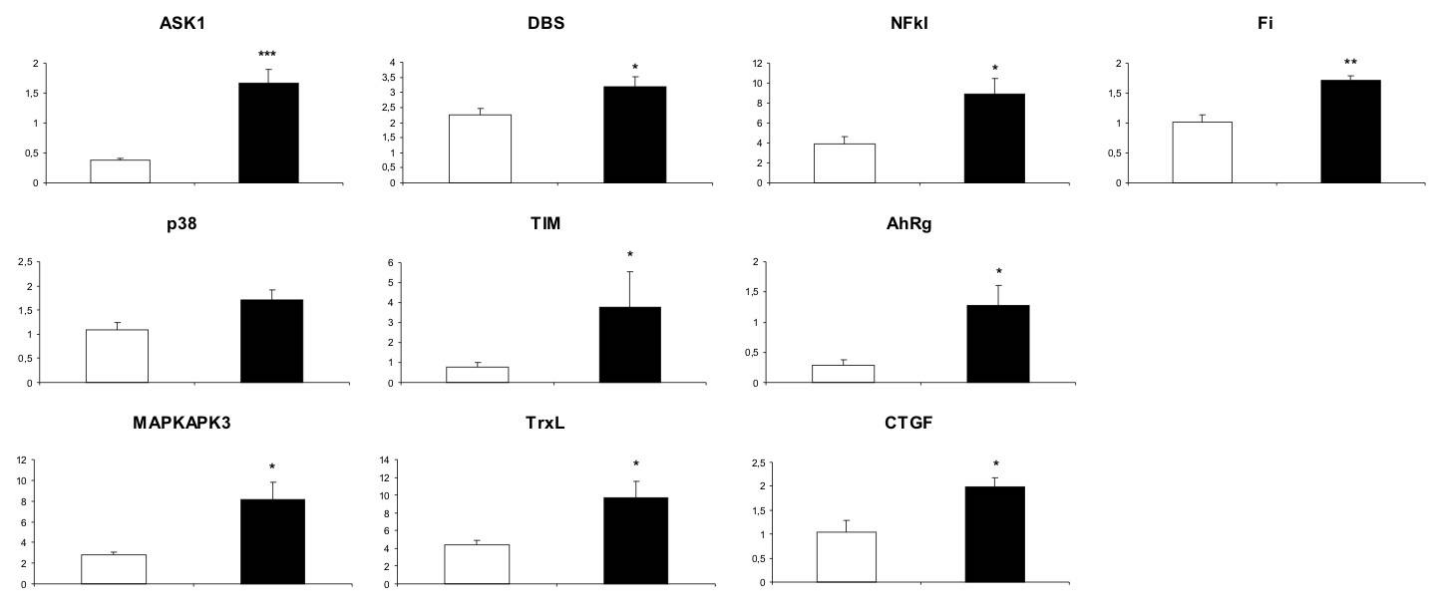
**Differentially expressed genes in Early M74-group tested with Q-RTPCR**. * = p < 0.05, ** = p < 0.01, *** = p < 0.001. White bars represent healthy and black bars M74 fry.

Selected E-group specific genes are presented in Fig. 5. Differential expression of several of these genes was verified using biological replication and quantitative real-time PCR (Q-RTPCR) (Fig. 6). A large number of these genes are stress-inducible and/or involved in apoptosis, signal transduction and formation of extracellular matrix. Notably, the expression of genes in corresponding Gene Ontology functional classes (e.g. 'response to stress', 'apoptosis', 'small GTPase mediated signal transduction', 'extracellular matrix') is significantly increased in the E-group compared to other M74 groups (Fig. 2).

Heat shock proteins (heat shock cognate 71 and heat shock protein 75) are upregulated in the E-group, along with STAT3, which can be induced by oxidative stress [52], and perforin1, a protein that causes the lysis of a variety of target cells (Gene Ontology Consortium). The role of oxidative stress in the development of early-onset mortality is further indicated by the observation that components of the stress-related mitogen activated protein kinase (MAPK)-pathway, ASK1, p38δ (differentially expressed in microarray experiments, Q-RTPCR p = 0.054) and MAPKAPK3, are upregulated in E 50. The apoptosis signal regulating kinase -1, ASK1, directly phosphorylates and activates the downstream kinases MKK4/7 and MKK3/6 [53]. MKK4/7 and MKK3/6 further phosphorylate and activate the downstream kinases JNK and p38. MAPKAPK3 (mitogen activated protein kinase activated protein kinase-3) is activated by JNK and p38 kinases [54]. In addition, the thioredoxin-like1 and 4A genes are upregulated.

ASK1, and its interaction with thioredoxin (Trx), is essential for apoptosis induced by, e.g., oxidative stress [55]. ASK1 forms an inactive complex with reduced Trx. Reactive oxygen species generated in oxidative stress oxidize Trx, which consequently dissociates from ASK, and activates it by inducing oligomerization and subsequent phosphorylation. Therefore, the Trx-ASK system serves as a molecular switch that converts a redox signal to a kinase cascade signal [55].

The guanine nucleotide exchange factors (GEF), DBS (DBL's big sister) and Rho guanine nucleotide exchange factor 5 (guanine nucleotide regulatory protein TIM) are upregulated in the E-group. Rho GTPases are part of the Ras-family of monomeric GTPases. They relay signals from receptor tyrosine kinases to the nucleus to stimulate cell proliferation and differentiation. Rho-related protein function has been proposed to integrate extracellular signals with specific targets regulating cell morphology, cell aggregation, tissue polarity, cell motility and cytokinesis [56]. DBS is a Rho-GEF belonging to the Dbl family. DBS stimulates dissociation of GDP from the Rho GTPases Rac1, RhoA and Cdc42, and promotes formation of Rho-GTP complex. It potentially links pathways that signal through Rac1, RhoA and Cdc42 [56]. Interestingly, JNK-MAPKKKs are also activated by GTP-binding proteins of the Rho-family [57], and therefore upregulation of Rho-GEF DBS and TIM may be related to this enhanced stress signalling event. TIM also belongs to the same family of Rho-GEFs containing a Dbl-Homology (DH) motif, but has been far less studied (Gene Ontology Consortium).

Since enhanced expression of these genes in E50 fry was detected by both microarray and Q-RTPCR, the effects on cell proliferation and cell death during early-onset M74 are clear. This suggestion is further strengthened by the upregulation of ceramidase. Ceramide is involved in signalling pathways of apoptosis, cell senescence, cell cycle and differentiation. Generally, ceramide is generated during apoptosis. Deacylation of ceramide by ceramidases yields sphingosine, which promotes apoptosis, induces cell cycle arrest and prevents proliferation [58].

The formation of tissues depends critically upon cell-to-cell contacts and the properties of the extracellular matrix (ECM). The upregulation of both ECM signalling and structural compounds in the E-group compared to healthy control fry and other M74 groups indicates disturbances in the formation and properties of the ECM and cell-to-cell contacts: genes for fibronectin, its receptor integrin beta-1, connective tissue growth factor (CTGF) and collagen alpha I chain are upregulated. In addition, collagen-binding protein 1 (47 kDa heat shock protein, colligin1) and tissue factor pathway inhibitor 2 precursor (TFPI-2) are upregulated and plasminogen precursor is downregulated, indicating an effort to prevent the degradation of ECM. TFPI-2 is a serine protease inhibitor that directly and indirectly regulates matrix proteolysis and connective tissue turnover [59].

ECM-cell interactions are also involved in the tissue response to injury. Fibroblasts migrate into wounds, proliferate, and produce large amounts of collagenous matrix, which helps to isolate and repair the damaged tissue [60]. It is possible that in the E-group, the stress is so severe that dying cells cause tissue injury, followed by fibroblast proliferation and ECM secretion.

Aryl hydrocarbon receptor γ (AhRg) is upregulated in the E-group. Most studies of AhR have centred on its role in, especially, dioxin toxicity [61]. However, recent studies on *C. elegans *and zebrafish embryos have indicated that it has an important role in neural development [62,63]. Thus, the result may associate the disturbance in AhR function to the neurological responses observed in M74. Our earlier results indicate that the DNA-binding activity of AhR may be reduced in M74-affected fry comparable to the I-group of this study [21], suggesting the possibility that mRNA levels and protein levels and activities are markedly different among M74 fry dying at different stages of development.

### The group developing symptoms most slowly shows gene expression changes compatible with disturbances in cell cycle and proliferation when clinical responses are seen

**Figure 7:**
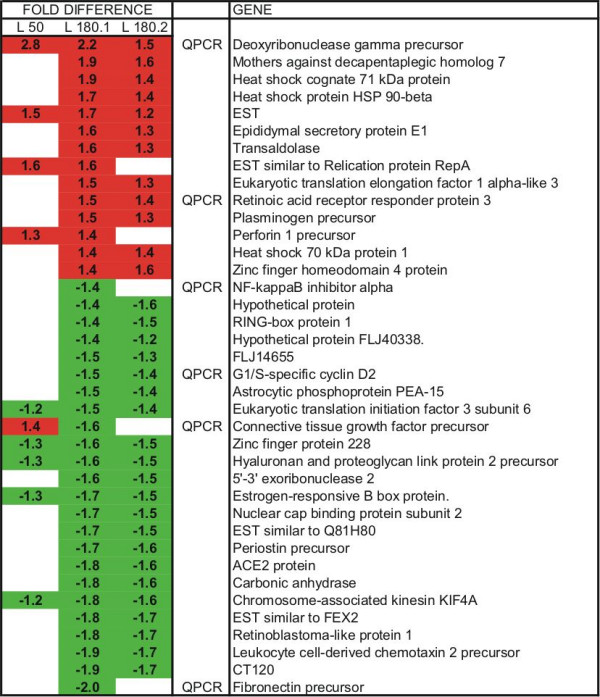
**Differentially expressed genes in Late M74-group**. L180.1 and L180.2 are biological replicates. All hybridizations are M74 vs healthy fry of the same age. Red indicates upregulation and green downregulation. The values are expressed as fold difference. White means that the expression ratio did not significantly deviate from unity. The genes analysed by Q-RTPCR are marked in a separate column.

**Figure 8:**
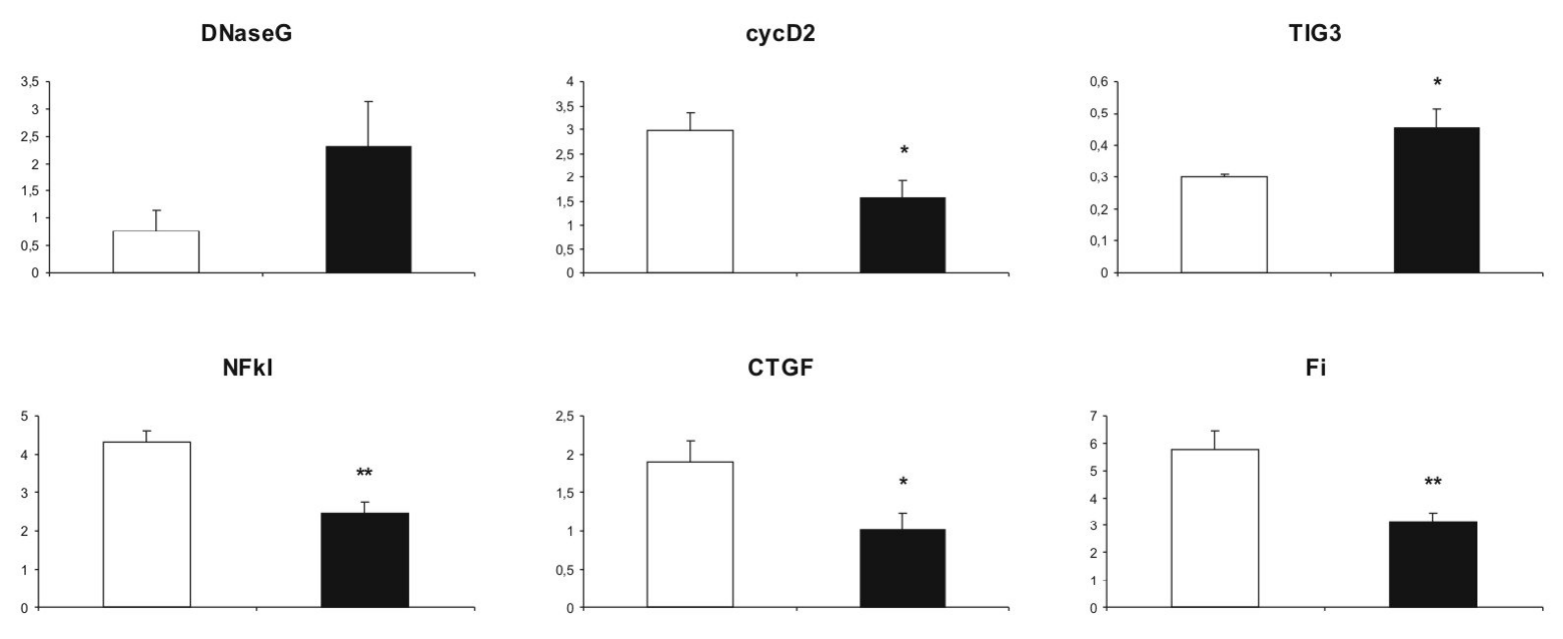
**Differentially expressed genes in Late M74-group tested with Q-RTPCR**. * = p < 0.05, ** = p < 0.01. White bars represent healthy and black bars M74 fry.

Selected L-group specific genes are presented in Fig. 7. Differential expression of several genes in this group was verified by biological replication and Q-RTPCR (Fig. 8). A number of genes showed differential expression only in the L-group, which develops M74 syndrome most slowly and usually shows only partial mortality. Some of these genes are related to stress, for instance small HSP, crystallin C and genes coding for HSP70 and HSP90 group proteins. It is likely that the stress is mainly oxidative, since other genes affected, such as IκBα, are members or targets of redox-sensitive signalling pathways. The clinical stage of the L-group (L180) also shows disturbances in cell cycle and chromatin-associated genes. In addition to the commonly affected genes, retinoic acid receptor responder protein 3 (TIG3) is upregulated in L180. TIG3 is regulated by retinoic acid and may mediate some of the growth suppressive effects of retinoids. The TIG3 fusion proteins exhibit growth suppressive and apoptosis-inducing activities in cells [64]. Moreover, G1/S-specific cyclin D2, which is expressed at entry to cell cycle, and kinesin KIF4A, which is required for mitotic chromosomal positioning and bipolar spindle stabilization (Gene Ontology Consortium), are downregulated. The hypothetical protein Q9BRX5, which may be associated with DNA replication, and an EST that is similar to Q81H80 and may be a member of the C-5 cytosine-specific DNA methylases (Gene Ontology Consortium), are also downregulated. In addition, many genes involved in the proper development of cell-to-cell contacts and extracellular matrix are affected in the L-group. For example, fibronectin and connective tissue growth factor are downregulated in L180. On the other hand, the gene for plasminogen, an extracellular matrix degrading enzyme, is upregulated.

Early in development, the L-group already shows marked upregulation of DNAse γ and cathepsin z, suggesting between-group differences in patterns of cell death among M74 fry. The L-group may be affected by a general decrease in metabolic rate, since genes associated with transcription, RNA processing and translation (CBP20, hypothetical protein Q9H0D6, hypothetical protein FLJ40338, eIF3e, hypothetical protein FLJ14655) are downregulated at the clinical stage. The results also show upregulation of an EST similar to SMAD7, which may have an important role in early haematopoiesis [65]. This finding is compatible with the general observation that an important component of erythrocyte development, globin gene expression, is disturbed.

## Conclusion

We suggest that the underlying cause of the M74 symptoms during the development of yolk-sac fry of Baltic salmon is oxidative stress. This suggestion is supported by altered expression of many redox-sensitive genes in the different groups. It is also compatible with our earlier observations on disturbances in the function of HIF-1α [21]. Oxidative stress seems to affect the fry differently, and the affected fry can be divided into three major groups with early, intermediate or late onset of the syndrome. If the disturbance occurs early, i.e. death occurs during the first third of the yolk-sac stage, virtually all the responses are compatible with an immediate stress that rapidly leads to the common terminal responses. If death occurs during the second third of the yolk sac stage (intermediate group), the terminal stage is preceded by a clear disturbance in globin gene expression, which will lead to internal hypoxia, when the animals grow and shift from predominantly skin-breathing to gill-breathing. In the absence of compensation for a reduced oxygen delivery, this group will then proceed to the terminal responses. The group developing M74 most slowly appears to compensate for a reduced oxygen delivery by slowing down metabolism, hence some fry escape death.

Since some of the genes differentially expressed in M74 fry are evolutionarily conserved, developmental disturbances in Baltic salmon can serve as a model for developmental disturbances and environmental stress responses in vertebrates in general.

## Methods

### Sample material

Atlantic salmon (*Salmo salar*), ascending Rivers Tornionjoki and Simojoki to spawn during the autumn of 2002 after feeding migration in the Baltic Sea, were caught by personnel of the Finnish Game and Fisheries Research Institute (FGFRI) for annual renewal of hatchery salmon stocks and monitoring of M74 incidence. Eggs were stripped from ready-to-spawn females and fertilized according to routine hatchery practice. The fertilized eggs were incubated over winter in female-specific groups (family groups, eggs from the same female) in the FGFRI hatchery at Lautiosaari (T = ca. 0.2°C), and in spring were transported to the FGFRI laboratory in Helsinki, where the yolk-sac fry of the studied groups developed (T = 4–7°C). Accumulated temperature units (ATU, degree-days) from hatching were used as the measure of yolk-sac fry development, since the development of fish eggs and larvae is temperature-dependent [66]. Thus, the use of ATU as a measure ensures that the fry are compared at similar developmental stages. Initially, family groups of healthy fry and fry suffering from M74 were chosen for sampling on the basis of egg thiamine content. The thiamine concentration in eggs developing into healthy fry in the study exceeded 1.0 nmol/g, and that in eggs developing into M74-affected fry was below 0.5 nmol/g. M74 groups were divided into early (E), intermediate1 (I1), intermediate2 (I2), intermediate3 (I3) and late (L) development of the syndrome (approximately 50 % mortality by 100–120 (E), 180–200 (I1 and I2), 225–250 (I3), and after 300 ATU (L)). Samples taken from preclinical, clinical and terminal stages of the syndrome (Table 1) were frozen in liquid nitrogen and stored at -80°C.

### Microarray analyses

We used a high-density cDNA microarray containing 1380 non-redundant salmonid clones. Clones were selected using two different sources: (1) EST (random clones) from common and subtracted cDNA libraries and (2) genes chosen by GO functional classes [14]. The array included clones originating from both rainbow trout (*Oncorhynchus mykiss*) and Baltic salmon (*Salmo salar*). Cross-species hybridization performances on salmonid 7.36 k cDNA microarray have been shown to be similar within the family *Salmonidae *(which includes rainbow trout and Atlantic salmon) [67]. An overview of the design and preparation of the microarray is given in [additional file 1] and the sequences of the printed clones in [additional file 2].

**Figure 9:**
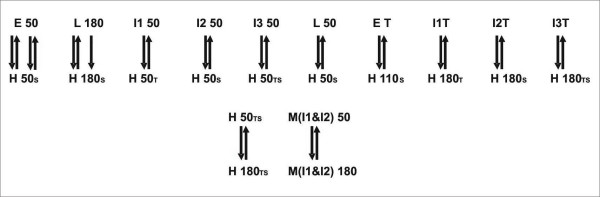
**Simplified scheme of all hybridizations made (27)**. S or T denotes River Simojoki or Torniojoki. For explanation of sample groups, see Table I.

Total RNA was isolated from whole fry using TRI-reagent (Sigma) according to the manufacturer's instructions. An additional purification of the extracted RNA was performed using RNeasy (Qiagen) according to the manufacturer's instructions, and the quality of RNA was monitored spectrophotometrically using the 260/280 nm OD ratio and 1% agarose-TBE (Tris-boric acid-EDTA) gels. In most analyses we compared healthy and M74 individuals (H/M). Every pool of three M74 fry was hybridized with a pool of three healthy fry of exactly same age and river of origin. However, two hybridizations were made to compare 50 ATU to 180 ATU healthy fry samples (H), and 50 ATU to 180 ATU M74 fry samples (M). Six individuals were pooled for these 50 vs 180 ATU comparisons. The hybridizations are represented schematically in Fig. 9. RNA (20 μg) was labelled with Cy3- and Cy5-dCTP (Amersham Biosciences) using Superscript II reverse transcriptase (Invitrogen) and oligo(dT)_12–18 _primer. cDNA was purified using Microcon YM30 (Millipore). We used a dye-swap experimental design except for the biological replicate of L180 (L180.2). For the first slide, the test (M74) and control (healthy) cDNAs were labelled in the order Cy5 and Cy3. For the second array the labelling order was reversed. The slides were pretreated with 1% BSA (fraction V), 5 × SSC and 0.1% SDS for 30 min at 50°C, followed by washes with 2 × SSC for 3 min and 0.2 × SSC for 3 min. The samples were hybridized overnight at 65°C in a mixture containing 1.3 × Denhardt's solution, 3 × SSC, 0.3 % SDS, 0.5 × DIG blocking buffer (Roche), 20 μg polyA and 42 μg yeast tRNA in a total volume of 80 μl. In total, 27 slides were used in this study. Scanning was performed with a ScanArray 5000 and images were processed by QuantArray (GSI Luminomics). The measurements of the spots were filtered by the criteria *I*/*B *= 3 *and *(*I*-*B*)/(*SI*+*SB*) = 0.6, where *I *and *B *are the mean signal and background intensities and SI, SB are the standard deviations. After subtraction of the mean background, LOWESS normalization was performed. Differential expression was analyzed by Student's t-test and false discovery rate (Q-values) according to Storey and Tibshirani [68]. The Q-values of the genes shown in Figs. 3, 4, 5, 6, 7 are listed in [additional file 3]. The genes presented in Figs. 4, 5, 6, 7, 8, 9 showed log_2 _expression ratios (ER) ≥ |0.6| at p < 0.05 in several samples. Expression values are means of 6 × 2 = 12 technical replicates. The GEO code for the microarray data is GSE2264.

For clustering and comparison of groups by functional categories, 590 genes with differential expression (p < 0.001) in at least one sample were chosen. For clustering we excluded genes that were measured in fewer than 75% samples, which reduced the number to 530. Euclidian distances between log_2_(ER) values were clustered by Ward's method. Log_2 _(ER) values in GO classes that included at least 5 genes were analysed using ANOVA and the Newman-Keuls test.

### Quantitative RT-PCR (Q-RTPCR)

The samples used in Q-RTPCR were from the same fry as those used for the microarray hybridizations. Total RNA (3 μg) was reverse transcribed using Powerscript (BD Biosciences) and oligo(dT)_12–18 _primer as described by the manufacturer. The 5- or 10- fold diluted cDNA (1 μl) was used as a template in a 25 μl PCR reaction containing 0.075 – 0.15 μM each of the forward and reverse gene-specific primers and Absolute QPCR SYBR Green mix (ABgene). All primers except AhRγ [69] were designed using Primer3 software. Amplicon sizes were 200–320 base pairs (bp), except for AhRγ, which was 482 bp. Gene names and forward and reverse primer sequences are listed in Table 2. PCR amplification was conducted on an Applied Biosystems PRISM 5700 Sequence Detection System. The PCR cycling conditions were as follows: initial denaturation and enzyme activation for 15 min at 95°C, followed by 30 cycles at 95°C for 15 s, at 64°C for 30 s and at 72°C for 30 s. Relative standards of Ultra FreeDA (Millipore) purified PCR products of known relative dilutions were made for each transcript. The copy number of each sample was standardized to 60S ribosomal L32 gene. The number of individual fry used for Q-RTPCR analysis was 6 for the Early group (at 50 ATU) and 5 for the Late group (at 180 ATU). Real-time PCR parametric data were analysed by the t-test, and non-parametric data by the Mann-Whitney U-test.

## Authors' contributions

KAMV participated in preparation of the microarray, carried out the sampling, sample preparation and microarray experiments and drafted the manuscript. PK participated in preparation of the microarray and samples. AK and HK designed and prepared the microarray. AK and SA performed the statistical analyses. PJV was responsible for the sample material. MN coordinated the research. All authors read and approved the final manuscript.

## Supplementary Material

Additional File 1 The design and preparation of the microarray. Click here for file

Additional File 2 The sequences of the printed clones. Click here for file

Additional File 3 Q-value listing of the genes presented in the figures 3, 4, 5, 7. Click here for file

## Figures and Tables

**Table 1:** Sample categories and their abbreviations, sample group's river of origin, and stage of M74 at the time sample was taken. The design for all E, I1, I2, I3 and L hybridizations was M74 vs healthy (M/H). In H and M hybridizations, healthy fry of 50 vs 180 ATU (H) and M74 fry of 50 vs 180 ATU were compared (M). Hybridization scheme is shown in Fig. 9.

Category	River of origin	50 ATU	110 ATU	180 ATU
Early (E)	Simojoki	Clinical (E 50)	Terminal (E T)	
Intermediate1 (I1)	Tornionjoki	Preclinical (I1 50)	not sampled	Terminal (I1 T)
Intermediate2 (I2)	Simojoki	Preclinical (I2 50)	not sampled	Terminal (I2 T)
Intermediate3 (I3)	Simojoki, Tornionjoki	Preclinical (I3 50)	not sampled	Clinical/Terminal (I3 T)
Late (L)	Simojoki	Preclinical (L 50)	not sampled	Clinical (L 180)
Healthy (H)	Simojoki, Tornionjoki	Healthy		Healthy
M74 (M)	Simojoki, Tornionjoki	Preclinical		Terminal

**Table 2:** Quantitative RT-PCR primer sequences and gene names.

Gene	Name	Sequence (5'-3')
60S ribosomal protein L32	60SL32Fw	GCAGGCGGTTTAAGGGTCAG
	60SL32Rv	AGTCTGGCATTGGGGTTGGT
Fibronectin (variant 3)	Fi Fw	GAGAACCCCCTGCACACTCA
	Fi Rv	TCGACATGGCAGCTACACCA
Connective tissue growth factor	CTGF Fw	TTTCAACCCCCAGTACGGAAA
	CTGF Rv	TGATTCCTTCCTTGCAACCTGA
NF-kappaB inhibitor alpha	IKBa Fw	CTCCTGAAGGCTGGCTGTGA
	IKBa Rv	CGGCCATTACACTGCTCCTG
Deoxyribonuclease gamma	DnaseG Fw	CGTGCCAAACTCTGCCAAAC
	DnaseG Rv	CATCCGCATCTTCCTCACCA
Thioredoxin-like protein	TrxL Fw	GGACTCGGACATTCCCAAGG
	TrxL Rv	AGTCCATGGAGCGAGGAAGG
MEK kinase 5 (ASK-1)	ASK1 Fw	CTGCCCATTGTGGAGTCAGG
	ASK1 Rv	AGGAGGCTGCTCAGGGTCAC
MAP kinase p38 delta	p38 Fw	TCGCCCTGCCATACTTCTCA
	P38 Rv	GAGGACCCCCACACACACAT
MAP kinase activated protein kinase-3	MAPKAPK3 Fw	GAGGATGACCATTGGGCAGTT
	MAPKAPK3 Rv	CAGCGGGTTGTTGGATGTGT
Guanine nucleotide exchange factor DBS	DBS Fw	TGCTGTGGGCATCACTGAGA
	DBS Rv	TCATGCTGCTCTCCCCTCTTC
Guanine nucleotide exchange factor TIM	TIM Fw	AGGAGGTCCAGGCCATCAAA
	TIM Rv	TCTTTGTGCAGCGACAGCAG
Aryl hydrocarbon receptor gamma	AhRg Fw	GTGGATGGGGTCACGTTCTCC
	AhR Rv	TGGGGGTGGTCTATGGTACCATC
Cyclin D2	cycD2 Fw	GAAGAGGTTTTTCCCTTGGCAAT
	cycD2 Rv	AACTTCTCCTCGGGCAATGG
Retinoid inducible gene 1	TIG3 Fw	AGGAAGCCCAGGCCTATGTG
	TIG3 Rv	TGATTGGCTGCTCAGGAAGC
