# Supplementary material for: Developmental disturbances in early life stage mortality (M74) of Baltic salmon fry as studied by changes in gene expression
Source: BMC Genomics. 2006 Mar 17;7:56. doi: 10.1186/1471-2164-7-56 (PMC1435884; doi:10.1186/1471-2164-7-56)
Supplement: Additional File 1 — The design and preparation of the microarray. [file 1471-2164-7-56-S1.doc]

**Design and preparation of microarrays**

The high-density cDNA microarray contained 1380 non-redundant salmonid clones. We used clones from two different sources 1) EST (random clones) from common and subtracted cDNA libraries prepared by the authors (KAMV and HK) and 2) genes chosen based on the functional classes of gene ontology (GO). The design and preparation of the microarray is described in detail in Krasnov et al.[1]. In brief, the platform was targeted for studies of acute conditions; it included 315 genes selected according to the Gene Ontology functional categories (e.g. stress and defense responses, regulation of cell cycle, signal transduction, chaperone activity and apoptosis). Of these, 282 clones were from the normalized multi-tissue library [2] and the rest were produced with RT PCR. Numeric representation of Gene Ontology functional categories, including all libraries and selected clones in the microarray are given in [1], Table 2. Results reported in [1] suggested that this was a suitable approach for designing the microarray as the numbers of differentially expressed genes are consistently higher among ESTs, while GO selection provides good coverage of the key functional classes. The array included clones originating from both rainbow trout (*Oncorhynchus* *mykiss*) and Baltic salmon (*Salmo salar*). Cross-species hybridization performances on salmonid 7.36 k cDNA microarrays have been shown to be similar within the family *Salmonidae* that includes rainbow trout and atlantic salmon [3].

Subtracted cDNA libraries were prepared from stressed rainbow trout (whole fry, brain, kidney and spleen of 1-year old fish) and M74-affected Baltic salmon whole yolk-sac fry at different stages of the disease (preclinical or terminal whole fry). Supression Subtractive Hybridization (SSH) cloning was performed as described in Diatchenko et al. [4]. Briefly, RNA was extracted with Trizol reagent (Invitrogen) and mRNA was purified with Dynabeads kit (Dynal). Tester and driver cDNA synthesis was performed with PowerScript reverse transcriptase (Clontech) and double-stranded cDNA was generated using Advantage DNA polymerase mix (Clontech). Ds cDNA was purified with phenol:chloroform:isoamylalcohol (25:24:1) – extraction. Tester and driver cDNAs were digested with RsaI. Digested cDNAs were purified with phenol:chloroform:isoamylalcohol (25:24:1) – extraction. The tester cDNA was subdivided into two portions (T1, T2), and each was ligated with a different adapter o/n at 16^o^C using a DNA ligase (New England Biolabs). T1 and T2 were hybridized with the driver first for 8 h at 68 ^o^C. This was followed by a second hybridization with fresh driver o/n at 68 ^o^C. First and second round PCR amplifications of enriched differentially expressed sequences were performed with the following parameters: 1^st^ PCR 75 ^o^C for 5 min, optimized number of cycles at (94 ^o^C for 30 sec, 66 ^o^C for 30 sec, 72 ^o^C for 1.5 min) 2^nd^ PCR optimized number of cycles at (94 ^o^C for 30 sec, 68 ^o^C for 30 sec, 72 ^o^C for 1.5 min) using Advantage DNA polymerase mix (Clontech). The products were cloned into TOPO (Invitrogen).

For the preparation of normalized libraries from stressed fish (whole fry, brain, kidney and spleen of 1-year old fish), synthesis of cDNA with PowerScript reverse transcriptase (Clontech) was primed with oligonucleotides including *EcoRI* and *NotI* sites. Double-stranded cDNA was generated using Advantage DNA polymerase mix (Clontech). The PCR products were purified with QIAquick kit (Qiagen), precipitated with ethanol and dissolved to 1 μg/μl in the hybridization buffer (1 M NaCl, 50 mM HEPES (pH 8.3), 1 mM EDTA). DNA was denaturated for 5 min at 94°C. Following re-association at 72°C for 16 hours, DNA was ethanol-precipitated and digested with 150 U of exonuclease III (MBI Fermentas) for 15 min at 37°C. This treatment eliminates re-associated double-stranded DNA. Single-stranded DNA was PCR amplified, size separated with agarose gel electrophoresis and cloned into pGEM®-11Zf (+) (Promega). The sequences were analysed with stand-alone blastn and blastx. Blastx search was made across all vertebrate proteins at cutoff e < -10. GEO annotation of platform (GPL1212) includes accession numbers of TIGR contigs and/or EST.

The cDNA inserts were amplified with PCR using universal primers and purified with a Millipore Montage PCR96 Cleanup Kit following the manufacturer’s instructions. DNA was spotted onto poly-(L) lysine-coated slides (Menzel) with Virtek ChipWriter Pro Arrayer in the Finnish DNA Microarray Centre at the Turku Centre of Biotechnology. The spotting buffer was DMSO. 8 spotting pens (Telechem) were in configuration of 2 x 4. The full array slide consisted of 12 array rows and 4 array columns. One array consisted of 13 spot rows and 14 spot columns. Spot diameter was 150 μm. Each slide consisted of 6 technical replicates of the 1380-clone array.

Reference List

1. Krasnov A, Koskinen H, Pehkonen P, Rexroad CE, III, Afanasyev S, Molsa H: **Gene expression in the brain and kidney of rainbow trout in response to handling stress.** *BMC Genomics* 2005, **6:**3.

2. Rexroad CE, III, Lee Y, Keele JW, Karamycheva S, Brown G, Koop B, Gahr SA, Palti Y, Quackenbush J: **Sequence analysis of a rainbow trout cDNA library and creation of a gene index.** *Cytogenet Genome Res* 2003, **102:**347-354.

3. Rise ML, von Schalburg KR, Brown GD, Mawer MA, Devlin RH, Kuipers N, Busby M, Beetz-Sargent M, Alberto R, Gibbs AR et al.: **Development and application of a salmonid EST database and cDNA microarray: data mining and interspecific hybridization characteristics.** *Genome Res* 2004, **14:**478-490.

4. Diatchenko L, Lau YF, Campbell AP, Chenchik A, Moqadam F, Huang B, Lukyanov S, Lukyanov K, Gurskaya N, Sverdlov ED et al.: **Suppression subtractive hybridization: a method for generating differentially regulated or tissue-specific cDNA probes and libraries.** *Proc Natl Acad Sci U S A* 1996, **93:**6025-6030.
